# Performance evaluation of generative pre-trained transformer on the National Veterinary Licensing Examination in Japan

**DOI:** 10.1038/s41598-026-37300-9

**Published:** 2026-02-16

**Authors:** Takahiro Kako, Daiki Kato, Takaaki Iguchi, Shiyu Qin, Miki Ando, Shoma Koseki, Hayato Shibahara, Haruka Motoi, Rin Isaka, Namiko Ikeda, Hiroto Toyoda, Takayuki Nakagawa

**Affiliations:** Laboratory of Veterinary Surgery, Graduate School of Agricultural and Life Sciences/Faculty of Agriculture, The University of Tokyo, 1-1-1 Yayoi, Bunkyo-ku, Tokyo, 113-8657 Japan

**Keywords:** Generative pre-trained transformer, Artificial intelligence, Large language model, Licensing examination, Japanese, Engineering, Mathematics and computing

## Abstract

Generative Pre-trained Transformer (GPT) models, which are large language models based on the transformer architecture, have enabled natural-language interaction with humans. GPT models have demonstrated high scores on National Medical Licensing Examination in various countries with translation. However, their performance on the National Veterinary Licensing Examination (NVLE) in Japan has not yet been explored. In this study, we evaluated GPT-4o, o1, and o3 on the 74th (2023) NVLE in Japan to compare the models, prompt designs (normal vs. optimized), and languages (Japanese vs. English). We then validated the best performance on the 75th (2024) and 76th (2025) NVLE using o3 with Japanese input and the normal prompt. As a result, o3 with Japanese input and the Normal prompt achieved the highest performance on the 74th NVLE, and both o1 and o3 outperformed GPT-4o. Furthermore, the validation tests using the 75th and 76th NVLE showed that o3 exceeded the minimum passing scoring rate in all sections, achieving an overall score of 92.9%. These findings indicate that recent GPT models can reliably answer the Japanese NVLE without requiring translation or elaborate prompt engineering, highlighting their potential as supportive tools in veterinary education and knowledge assistance in Japan.

## Introduction

Artificial Intelligence (AI) has been increasingly applied across medicine, including diagnosis^1,2^, treatment^3^, drug development^4^, and education^5–7^. Specifically, machine learning (ML), a subset of AI, learns patterns or rules from data without explicit programming^8^. The rapid growth of medical data has further accelerated the application of ML in this field ^9^. In particular, natural language processing (NLP) has attracted attention^10^, as it enables natural text-based communications with humans^11^. In the medical domain, NLP could be used as a supportive tool. In previous studies, NLP models summarized research findings and patients’ clinical records, and assisted in clinical decision-making^12,13^. The transformer architecture has recently reinforced the ability of NLP. The transformer is a neural network model that utilizes a self-attention mechanism to determine the importance of each word in understanding the meaning of a sentence, allowing it to learn and generate coherent text^14^.

Generative Pretrained Transformer (GPT) is a novel NLP model developed by OpenAI^15^, based on the transformer architecture. Several versions of GPT models have been released since the first GPT-1 model was introduced in 2018. GPT-4o is a widely used model mainly available through ChatGPT, which is a dialogue-style chatbot released in May 2024^16^. O1 is a cutting-edge model that thinks more deeply and logically with a chain-of-thought, and was released in December 2024^17,18^. O3 was released subsequently as a reinforced model of o1 in April 2025^19^. The main task of GPT is predicting the most reasonable next word, and GPT generates coherent text by repeating the prediction. Although GPT is not specifically fine-tuned for specific fields, such as human or veterinary medicine, GPT has demonstrated significant performance even in the medical domain^20,21^. This high performance has also been shown in the studies using the National Medical Licensing Examination (NMLE) in various countries such as the United States and Germany^22,23^. Particularly, GPT exceeded the minimum passing scoring rate on the Japanese NMLE with questions translated to English^24^. Moreover, regarding the veterinary field, GPT-4 achieved an improved score in the North American Veterinary Licensing Examination^25^. However, there is no study about the performance of GPT on the National Veterinary Licensing Examination (NVLE) in Japan.

Thus, we compared the models (GPT-4o, o1, o3), prompts, and languages using the 74th (2023) NVLE in Japan and validated the performance in correctly answering the veterinary questions of the 75th (2024) and 76th (2025) NVLE in Japan with the most favorable settings.

## Materials and methods

### Study overview

This study evaluated the performance of GPT models on the NVLE in Japan. For model comparison, the GPT-4o, o1, and o3 models (Open AI, Inc., San Francisco, CA, USA) were utilized. For prompt comparison, the Normal solving prompt and the Optimized solving prompt were generated. For language comparison, Japanese (original text), English (generated through the Normal translating prompt), and English (generated through the Optimized translating prompt) were utilized.

**Fig. 1:**
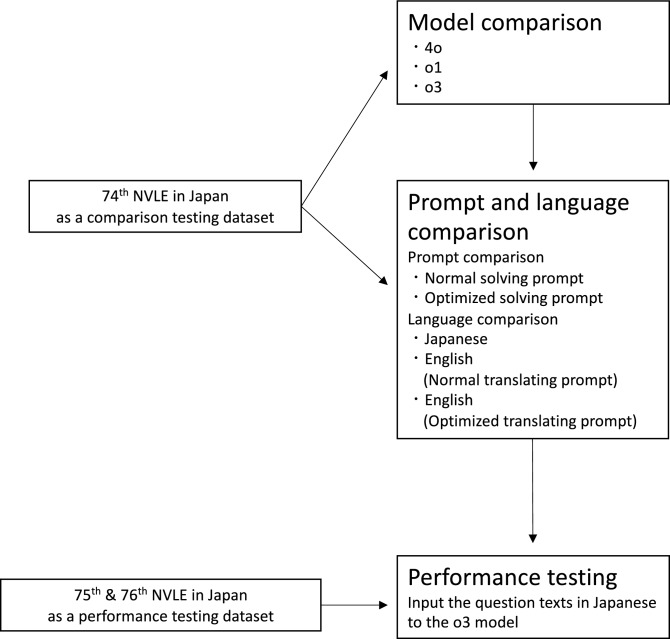
The study overview. The 74th NVLE in Japan was used as a comparison testing dataset. First, three GPT models (GPT-4o, o1, and o3) were compared. Subsequently, two prompts (the Normal solving prompt and the Optimized solving prompt) and three languages of the question (Japanese, English translated with Normal translating prompt, and English translated with Optimized translating prompt) were compared. Finally, the performance of GPT on the 75th and 76th NVLE in Japan with the most favorable settings (o3, Normal prompt, Japanese) was validated.

Initially, the questions from the 74th NVLE in Japan (February 2023) were used to clarify the differences in performance among models, prompts, and language settings in producing the correct answers. Subsequently, we assessed the performance of the best GPT model (o3) with the most favorable prompt conditions using the 75th and 76th NVLE in Japan (February 2024 and February 2025, respectively) (Fig. 1).

All analyses using GPT were performed under the supervision of human authors, who verified the accuracy of data processing and statistical procedures.

### National Veterinary Licensing Examination (NVLE) in Japan

The NVLE in Japan consists of five sections: Essential, A, B, C, and D, and the questions are all multiple-choice with five options. The Essential, A, and B sections contain questions based solely on text, whereas the C and D sections include questions that combine both text and images. In section D, 60 questions are organized into 30 pairs. Each pair is based on a single passage, such as a clinical case or scenario, which provides relevant information.

For the analysis of incorrect responses, all questions were categorized based on the official examination guidelines into four areas: Basic knowledge of veterinary medical practice, Fundamental veterinary science, Veterinary public health, and Clinical veterinary medicine.

The questions and answers for the 74th (2023), 75th (2024), and 76th (2025) NVLE in Japan were obtained from the official website of the Ministry of Agriculture, Forestry, and Fisheries (MAFF) of Japan^26^. The images of questions were obtained by scanning the official printed booklets with a Canon MF741C scanner at a resolution of 300 dpi and saved as PDF files without additional adjustments.

### Generative pretrained transformer

In this study, three models were used: GPT-4o (knowledge cutoff: October 1, 2023), o1 (knowledge cutoff: October 1, 2023), and o3 (knowledge cutoff: June 1, 2024). All models were accessed via the OpenAI API between April 15 and May 17, 2025.

### Data input method and evaluation of output data

The input and evaluation workflow were implemented using Python scripts. The question texts and multiple-choice options were stored in Microsoft Excel files, which were programmatically read and formatted into prompts for the OpenAI API.

The prompts were manually developed with reference to the previous report^24^. To enhance response accuracy, we developed the Optimized prompt by adding relevant contextual information, such as specifying that the examinations were conducted in Japan, instructing the model to engage in more deliberate reasoning, and guiding it to render technical content in language accessible to non-experts during translation.

For the English-language setting, the original Japanese questions and choices were first translated into English using the same GPT model that would later answer the questions via the API. The translated content was then incorporated into the prompts for subsequent model queries.

For the image-based questions, the PDF files containing the images are exported as JPEG files and then, the JPEG files are Base64 encoded and submitted to GPT via the OpenAI API.

The temperature was set to 1.0 for all experiments. For o1 and o3 models, the temperature is fixed at the default value of 1.0 and cannot be modified. To standardize the experimental conditions, we also fixed the temperature for GPT-4o to 1.0. Because a temperature of 1.0 introduces some randomness in the outputs, each question was submitted to the API three times to obtain a more stable estimate of model performance. Regarding the image-based questions, the same image file is submitted for each question without any adjustment. For each trial, the prompts instructed the model to return only the final answer choice (e.g., “1”, “2”, “3”, “4”, or “5”) without any explanation or reasoning. Answer correctness was determined by comparing each model’s output with the official answers published by the MAFF. For questions officially classified as ambiguous (i.e., those with multiple acceptable answers), all officially accepted answer choices were treated as correct. Questions officially classified as invalid (i.e., not scored in the official exam) were excluded from this study.

### Statistics

Statistical analyses were conducted using Cochran’s Q test and McNemar’s test on the mode (majority vote) of the correctness label derived from the three responses per question to evaluate model correctness. To control the family-wise error rate (FWER) in the pairwise comparisons of model performance, we applied multiplicity adjustments. For analyses with 3 pairwise comparisons (i.e., the model comparisons), we applied the Bonferroni correction, which is appropriate when the number of comparisons is small. For the analyses involving 15 pairwise comparisons (i.e., prompt and language comparisons), we applied the Holm correction, which is suitable for a large number of comparisons. All statistical calculations were performed with Python, using the statsmodels package (version 0.14.4).

## Results

### Comparison of the models on the 74th NVLE (2023)

**Fig. 2:**
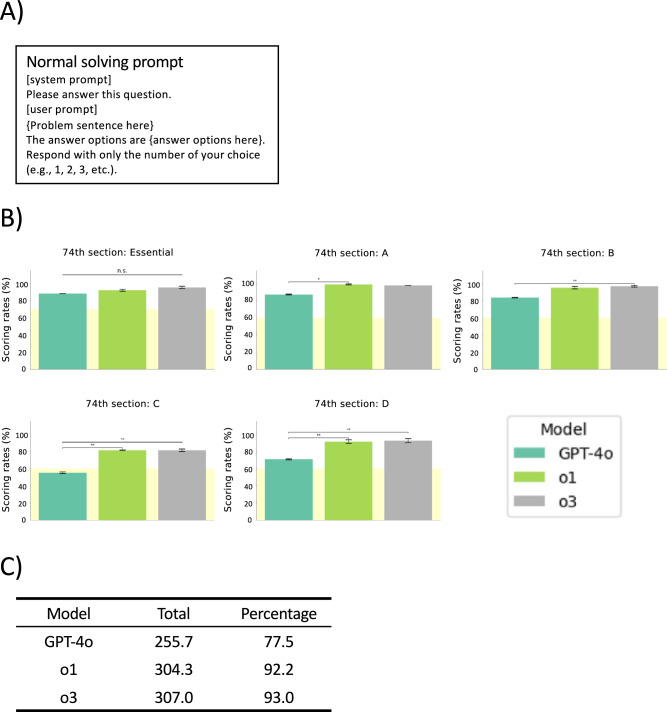
The model comparison tests. (**A**) The Normal solving prompt used in the model comparison tests. (**B**) Scores achieved by each model on the 74th NVLE in Japan. The yellow shading indicates the minimum passing scoring rate for each section (70% for the essential section and 60% for the other sections). Values are expressed as mean ± SD of triplicate samples. Statistical significance across three related groups was assessed using Cochran’s Q test, and pairwise comparisons were performed using McNemar’s test with Bonferroni correction. *p < 0.05, **p < 0.01. (**C**) Overall average score for each model and percentage of correct answers, out of 330 questions.

Firstly, the three GPT models (GPT-4o, o1, and o3) were compared on the questions from the 74th (2023) NVLE in Japan. The questions were fed into each model in Japanese with the normal solving prompt (Fig. 2A). The average percentages of correct answers of GPT-4o, o1, and o3 were 88% (44/50), 92% (46/50), 96% (48/50) in the Essential section, 87.1% (69.7/80), 98.8% (79/80), 97.5% (78/80) in the section A, 83.4% (66.7/80), 95% (76/80), 96.6% (77.3/80) in the section B, 55% (33/60), 81.2% (48.7/60), 81.2% (48.7/60) in the section C, and 70% (42.3/60), 91.2% (54.7/60), 92.2% (55.3/60) in the section D, respectively (Fig. 2B). We calculated the overall average percentage of correct answers for each model, and those were 77.5% (255.7/330) for GPT-4o, 92.2% (304.3/330) for o1, and 93% (307/330) for o3. O1 model outperformed GPT-4o in Sections A, C, and D, and o3 outperformed GPT-4o in Sections B, C, and D. Notably, in Section C, the GPT-4o did not reach the minimum passing scoring rate (60%), whereas o1 and o3 substantially exceeded the minimum passing scoring rate. Although no significant differences were observed between o1 and o3, o3 achieved a 0.6% higher overall score compared to o1 and therefore was selected for further experiments (Fig. 2C).

### Comparison of both prompts and languages on the 74th NVLE in Japan (2023)

**Fig. 3:**
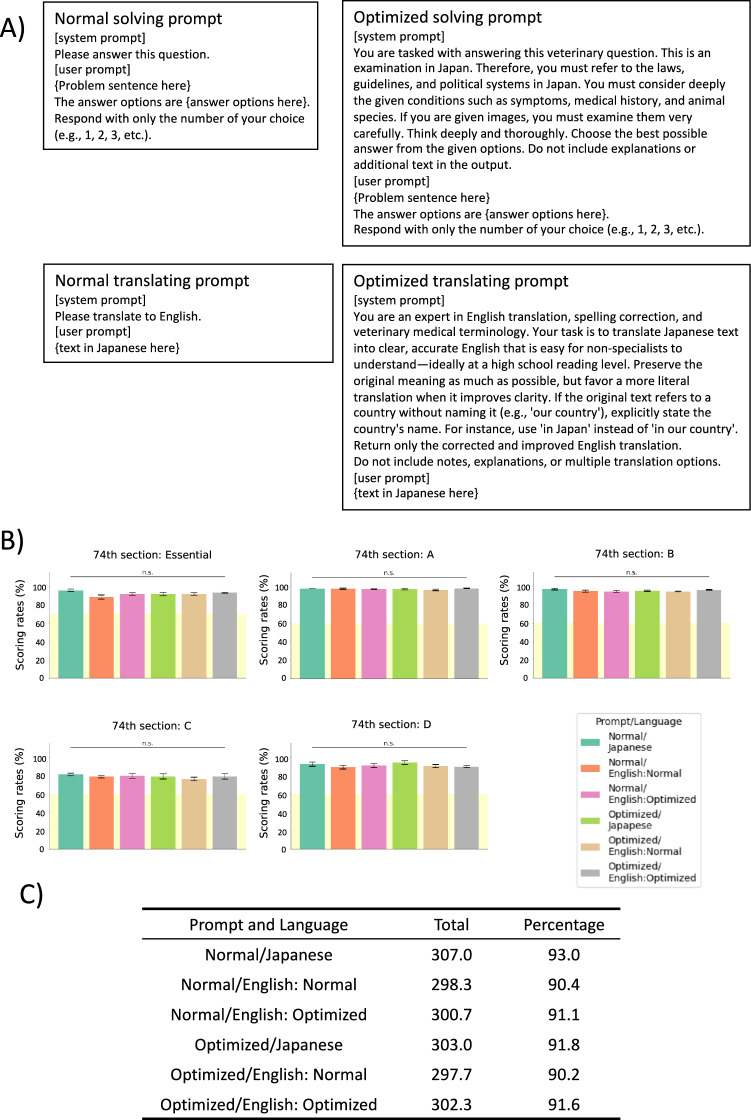
The prompts and languages comparison tests. A) The prompts used in the prompts and languages comparison tests. B) Scores achieved by o3 on the 74th NVLE in Japan in various settings. The yellow shading indicates the minimum passing scoring rate for each section (70% for the essential section and 60% for the other sections). Values are expressed as mean ± SD of triplicate samples. Statistical significance across six related groups was assessed using Cochran’s Q test, and pairwise comparisons were performed using McNemar’s test with Holm correction. ns, not significant (*p* > 0.05). C) Overall average score for each prompt and language condition and percentage of correct answers, out of 330 questions.

Next, the effects of both prompt design and question language on performance were examined on the questions from the 74th (2023) NVLE in Japan. There were six conditions in total (2 for solving prompt {the Normal solving prompt and the Optimized solving prompt} and 3 for language {Japanese, English translated with the Normal translating prompt, and English translated with the Optimized translating prompt}) (Fig. 3A). Interestingly, no significant difference was observed among the conditions, and O3 model surpassed the minimum passing scoring rate in any conditions (Fig. 3B,C). Therefore, the simplest condition, in which the questions were input in Japanese using the Normal solving prompt, was adopted for the subsequent analysis.

### GPT-o3 model performance on the 75th and 76th NVLE in Japan (2024, 2025)

**Fig. 4:**
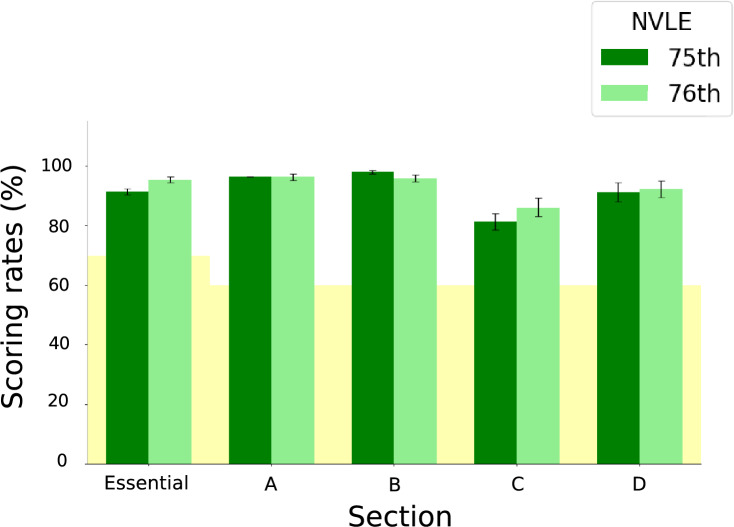
Performance validation tests on the 75th and 76th NVLE. Scores achieved by o3 on the 75th and 76th NVLE in Japan with Japanese original question text and the Normal solving prompt. The bars in dark green and in light green show o3’s scoring rate at each section in the 75th and 76th NVLE, respectively. The yellow shading indicates the minimum passing scoring rate for each Sect. (70% for the essential section and 60% for the other sections). Values are expressed as mean ± SD of triplicate samples.

Furthermore, we validated the performance of the o3 model in solving the questions from the 75th (2024) and 76th (2025) NVLEs in Japan under the simplest condition (i.e., inputting the Japanese original text using the Normal solving prompt). The average scores in the 75th NVLE were 91.4% (45.7/50), 96.3% (77/80), 97.9% (78.3/80), 81.4% (48/59), and 91.2% (54.7/60) for the Essential, A, B, C, and D sections, respectively. The corresponding scores in the 76th NVLE were 95.4% (47.7/50), 96.2% (76/79), 95.9% (76.7/80), 86.2% (51.7/60), and 91.7% (54.3/59), respectively. Regarding the variability of the individual outputs, we analyzed the o3 model’s responses across three iterations for each question. For the 75th and 76th NVLE (329 and 328 questions in total, respectively), 298 and 299 questions had identical outputs across all three iterations, 27 and 28 questions had two identical outputs and one differing output, and 4 and 1 questions showed completely different outputs across all three iterations. The overall scores were 92.3% (303.7/329) and 93.4% (306.4/328) for the 75th and 76th NVLEs, respectively. For both datasets, the o3 model achieved scores well above the minimum passing scoring rate (70% for the Essential section and 60% for the others) across all sections (Fig. 4).

### Analysis of incorrect responses

To investigate patterns in incorrect answers, we analyzed the questions that the o3 model answered incorrectly on the 75th and 76th NVLEs in Japan. We calculated the ratio of incorrect answers from o3 for each area categorized based on the official examination guidelines. The total number of incorrect answers from o3 was 141 out of 1971 (7.2%), and that of each area is 15 out of 120 (12.5%) for the Basic knowledge of veterinary medical practice, 21 out of 639 (3.3%) for the Fundamental veterinary science, 30 out of 531 (5.6%) for the Veterinary public health, and 75 out of 681 (11.0%) for the Clinical veterinary medicine. The Basic knowledge of veterinary medical practice and the Clinical veterinary medicine had higher error rates compared to the Fundamental Veterinary science and the Veterinary public health (Table 1).

**Table 1:** Summary of incorrect answers.

Category	Incorrect answer	Total question	Percentage
Basic knowledge of veterinary medical practice	15	120	12.5
Fundamental veterinary science	21	639	3.3
Veterinary public health	30	531	5.6
Clinical veterinary medicine	75	681	11.0

The number of incorrect answers for each area at the performance validation tests using the 75th and 76th NVLE in Japan, and their percentages out of the total number of questions.

## Discussion

In this study, we evaluated the performance of GPT models on the NVLE in Japan. In the model comparison tests using the 74th NVLE, all tested conditions except for GPT-4o in Section C exceeded the minimum passing scoring rate, and o1 and o3 outperformed GPT-4o. In the prompt and language comparison tests with o3, there was no significant difference in performance attributable to prompt formats or the language. Furthermore, in the validation tests using the 75th and 76th NVLE, o3 achieved scores well above the minimum passing scoring rate across all sections with the Normal solving prompt and the original Japanese questions.

In the model comparison tests, GPT-4o did not meet the minimum passing scoring rate for Section C, whereas o1 and o3 surpassed the minimum passing scoring rate in all sections. Notably, o1 and o3 demonstrated significantly improved performance compared to GPT-4o across most sections. O1 and o3 models have more sophisticated reasoning ability than GPT-4o^19,27,28^. This improvement in the reasoning ability is likely attributable to the better performance of o1 and o3 on the NVLE in Japan. However, no significant difference was observed between o1 and o3, which implies that the ability to answer the questions of degree-level veterinary medical examinations may have reached a ceiling.

Regarding prompt and language comparisons, o3 consistently exceeded the minimum passing scoring rate without requiring elaborate prompt engineering or translation into English. A recent study has reported that translating Japanese NMLE questions into English improved GPT-4o’s performance^24^. One possible explanation provided in the study is that the primary training data are mainly from English-language websites^29^, which may therefore enable GPT to understand English better than Japanese^30^. In contrast, our findings did not demonstrate improvements when translating the questions into English, indicating that GPT’s ability to comprehend Japanese has advanced to a level comparable to its ability to understand English, at least in the context of simple veterinary medical questions. These findings highlight the potential of GPT for more direct application in the veterinary medical field through the Japanese language.

All models showed the noticeable decline in accuracy on image-based questions in Sections C and D. This trend was consistent with a previous report evaluating the performance of LLMs in the veterinary field ^31^ and may reflect insufficient domain-specific training of current ViT-based image encoders on veterinary medical images. However, despite the difficulty in capturing information from domain-specific images, o1 and o3 demonstrated superior performance on the image-based questions in Sections C and D compared to GPT-4o. This result would reflect the improvement of image recognition and integration of visual and textual reasoning^18,19,27^. Notably, the score of GPT-4o for Section C fell below the minimum passing scoring rate, consistent with findings from the prior study of GPT-4o on the NMLE in Japan, in which GPT-4o also underperformed on the image-based questions compared with text-based questions. However, in our study, the score for Section C was much lower than that for image-based questions in the prior study on the NMLE in Japan, with a correct answer rate of 55% compared to 68.7% in the prior study^32^. Moreover, GPT-4o achieved a higher score in the previous study, even when solving image-based questions without access to images, compared to its performance on Section C with images in our study^24^. These findings suggest that the image-based questions in the NMLE in Japan were less reliant on visual information and could often be solved using textual context alone. In contrast, the NVLE in Japan, particularly Section C, relied so heavily on image information that the model’s image recognition capability directly influences performance. Compared to Section C, Section D includes extensive textual supplementary information, which likely contributed to GPT’s higher performance observed in Section D compared to Section C. Overall, o1 and o3 were able to achieve passing performance on the NVLE in Japan, which includes the highly image-dependent section, underscoring their enhanced capacity for visual-textual reasoning.

In the validation tests using the 75th and 76th NVLEs in Japan, o3 achieved the minimum passing scoring rate across all sections under the conditions of the Normal prompt with the original Japanese questions, recording accuracy rates of approximately 80% for Section C and over 90% for the other sections. Previous studies evaluating GPT-4o on the NMLE in Japan reported accuracy rates of around 80% for essential questions and about 70% for the other sections^24,32^. Furthermore, studies conducted on the performance of GPT-4o on NMLE in the US and China showed lower scores than those observed in this study^22,33^. Although we cannot directly compare the results of different examinations, o3 nevertheless achieved substantially higher scores than previously reported GPT models. In addition, because there are currently no published studies evaluating the performance of o3 on either the NVLE or the NMLE, direct comparisons with existing literature are not possible.

The analysis of incorrect responses revealed the trend of lower performance in the Basic knowledge of veterinary medical practice and the Clinical veterinary medicine areas compared to the other areas. The area of Basic knowledge of veterinary medical practice includes questions on veterinary laws, which likely contributed to the lower performance in this area. The questions regarding Japanese legal topics were particularly challenging for GPT^24^, as these questions often rely on the country-specific legal systems and frameworks, which are likely underrepresented in its predominantly English-language training dataset^29^. The Clinical veterinary medicine area was also challenging for GPT, as it requires not only factual knowledge but also the ability to integrate information from multiple sources and to perform multistep reasoning, both of which remain limited in current GPT models^34^. Thus, although GPT has demonstrated marked improvements in logical reasoning, the accurate resolution of such complex tasks would continue to pose a considerable challenge^19,27,28^.

It should be noted that OpenAI explicitly prohibits the direct use of GPT for medical or veterinary diagnosis and treatment decision-making^35^. Even when GPT is not directly used for such purposes, particular caution is warranted, as GPT may generate fabricated information (so-called hallucinations)^36^ and functions as an opaque black-box system^30^. Moreover, we caution that the medical benchmark scores do not directly reflect real-world readiness^37^. Therefore, realistic applications in clinical practice are limited to supportive roles, such as diagnostic assistance, risk detection, automatic summarization of medical records, and format conversion^38^. Moreover, GPT could also be effectively utilized in educational settings^5^, for example, as a virtual tutor for veterinary students. The demonstrated performance of GPT in this study indicates its reliable ability to assist in Japanese veterinary medicine and education.

This study contains several limitations. First, the 74th (2023) NVLE, which was used in the model comparison tests and the prompt and language comparison tests, was publicly available (March 2023) before the knowledge cutoffs of GPT-4o, o1, and o3 (October 2023, October 2023, and June 2024, respectively), therefore, we cannot completely exclude the possibility that the question sentences of the 74th NVLE were used in the training dataset of those models. Additionally, the 75th (2024) NVLE, which was used in the validation tests, was also publicly available (March 2024) before the knowledge cutoff of o3 (June 2024). Therefore, the results on the 74th and 75th NVLEs need to be interpreted with caution. The 76th (2025) NVLE was the only examination released after the knowledge cutoffs of GPT-4o, o1, and o3, and was therefore reserved exclusively for the validation tests. Because the 76th NVLE was unavailable for model training, the o3 model’s high performance is not solely attributable to data leakage and provides reliable evidence of the model’s innate ability. Therefore, since our claims are grounded in the 76th NVLE results, our study’s conclusions are valid and technically sound. Second, the temperature was fixed at 1.0 for this study because the temperature for o1 and o3 models cannot be modified from their default value of 1.0, and also set the temperature for GPT-4o to 1.0 in order to standardize the experimental conditions. In performance tests that primarily evaluate model knowledge, using a temperature of 0 or a low value is preferable, because a low temperature produces more deterministic outputs. It should also be noted that temperature may interact with sampling strategies such as majority voting, potentially influencing the stability or the accuracy. Although this interaction was not systematically evaluated in this study, it may be a relevant factor for future investigations. Finally, the performance of LLM is highly time-sensitive, as the models are frequently updated and their architectures and training data are not publicly disclosed. Therefore, the results of this study reflect the performance of the evaluated models at the time of experiment and may not fully generalize to future models. Future work could also explore differences in performance of state-of-the-art models, other architectures such as retrieval-augmented generation, and prompt generation methods such as automated prompt engineering.

In conclusion, o3 achieved high performance well above the minimum passing scoring rate across all sections using Japanese text input and the normal prompt. Notably, this performance was achieved without the need for translation or advanced prompt engineering, reflecting the substantial improvements in GPT’s inherent capabilities. These findings suggest that GPT’s evolution opens new possibilities for knowledge support in veterinary medicine.

## Data Availability

The code used in this study is publicly available at (https:/github.com/UTokyo-VetSurgeryLab/gpt-japanese-nvle-evaluation). Additional data on the results of this study are available from the author upon reasonable request.
